# Novel Uses of Radioactive Seeds in Surgical Oncology: A Case Series

**DOI:** 10.7759/cureus.5706

**Published:** 2019-09-20

**Authors:** Michèle Beniey, Kerianne Boulva, Ahmad Kaviani, Erica Patocskai

**Affiliations:** 1 Surgery, Centre Hospitalier de l'Université de Montréal (CHUM), Montreal, CAN

**Keywords:** radioactive seed, axilla, nodal metastases, loco-regional control

## Abstract

The localization of nonpalpable axillary metastatic lymph nodes has been achieved using several techniques in the past. Amongst these techniques, the use of radioactive iodine seeds is increasingly spread, and was initially reserved to breast-conserving surgery. Many studies have assessed the use of radioactive seed localization for the surgical management of breast cancer patients diagnosed with lymph node metastases. However, few articles have reported their utilization in other cancer subtypes and in complex clinical situations. This case series describes the innovative use of radioactive seeds in the axilla in five patients, including one case of squamous cell carcinoma skin cancer, one case of malignant melanoma, and three cases of invasive breast cancer.

## Introduction

Although breast-conserving surgery using radioactive seeds is a well-described procedure [1-4], it is only recently that surgical oncologists have started to use iodine-125 (I125) seeds for targeted node retrieval. Most studies have focused on the targeted identification of previously proven positive axillary lymph nodes post neoadjuvant chemotherapy. In fact, several methods have been published, including wire-guided localization [5], the MARI (Marking the Axilla with Radioactive Iodine seeds) procedure [6-8], intraoperative ultrasound guidance, and targeted axillary dissection [9].

Nevertheless, we wanted to extend radioactive seed localization to complex surgical oncology cases, in different clinical contexts than neoadjuvant chemotherapy. Here, we report using iodine-125 radioactive seeds to retrieve metastatic axillary nodes in a diverse array of complex cases.

This case series describes the use of I^125^ seeds as a key adjunct to solving challenging clinical situations in primary malignant melanoma, as well as recurrent squamous cell carcinoma of the skin and invasive breast cancer.

## Case presentation

Case 1

**Figure 1:**
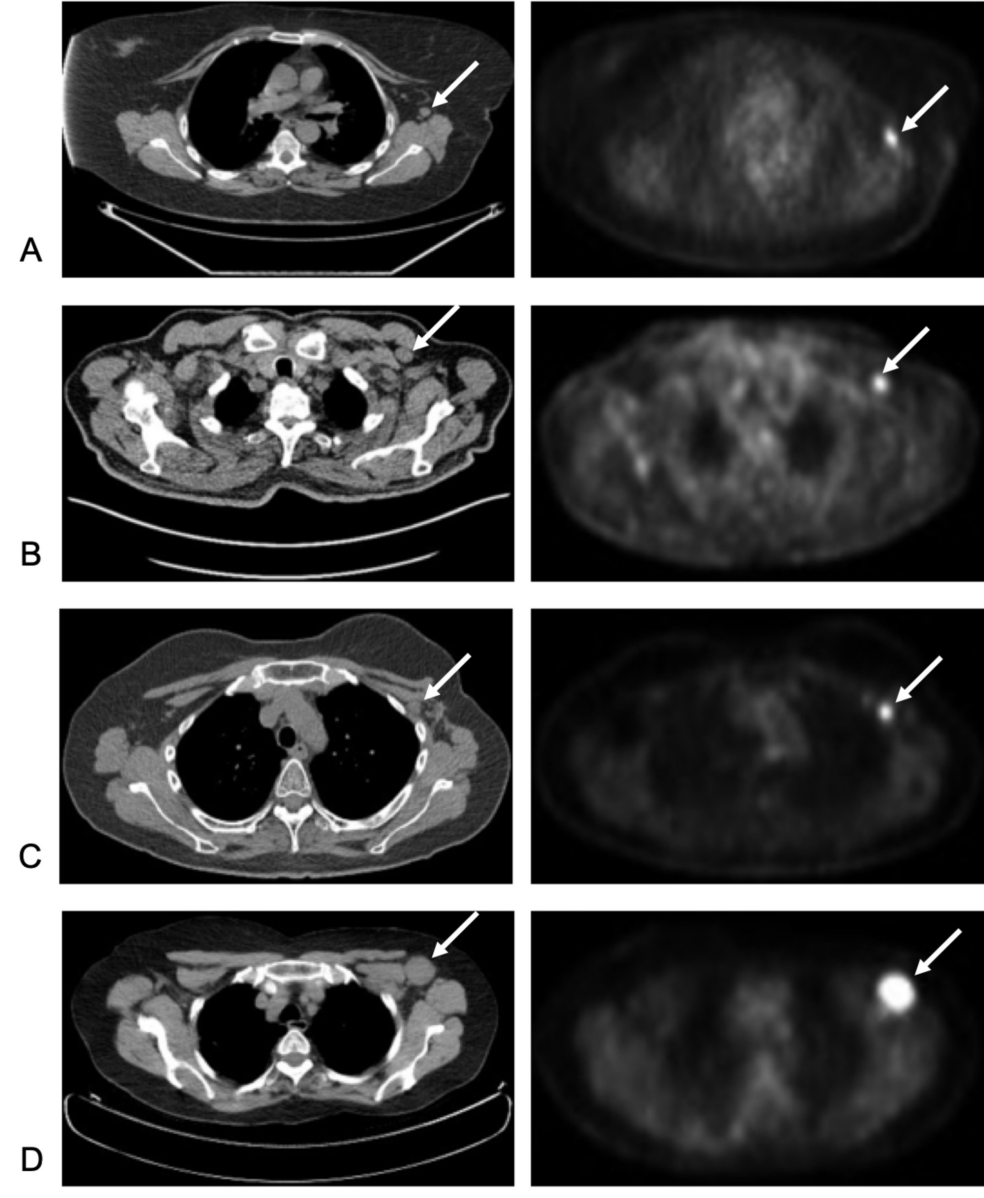
PET/CT images of A) case 1; B) case 2; C) case 3; and D) case 4. Arrows indicate the position of hypermetabolic lymphatic nodes.

**Figure 2:**
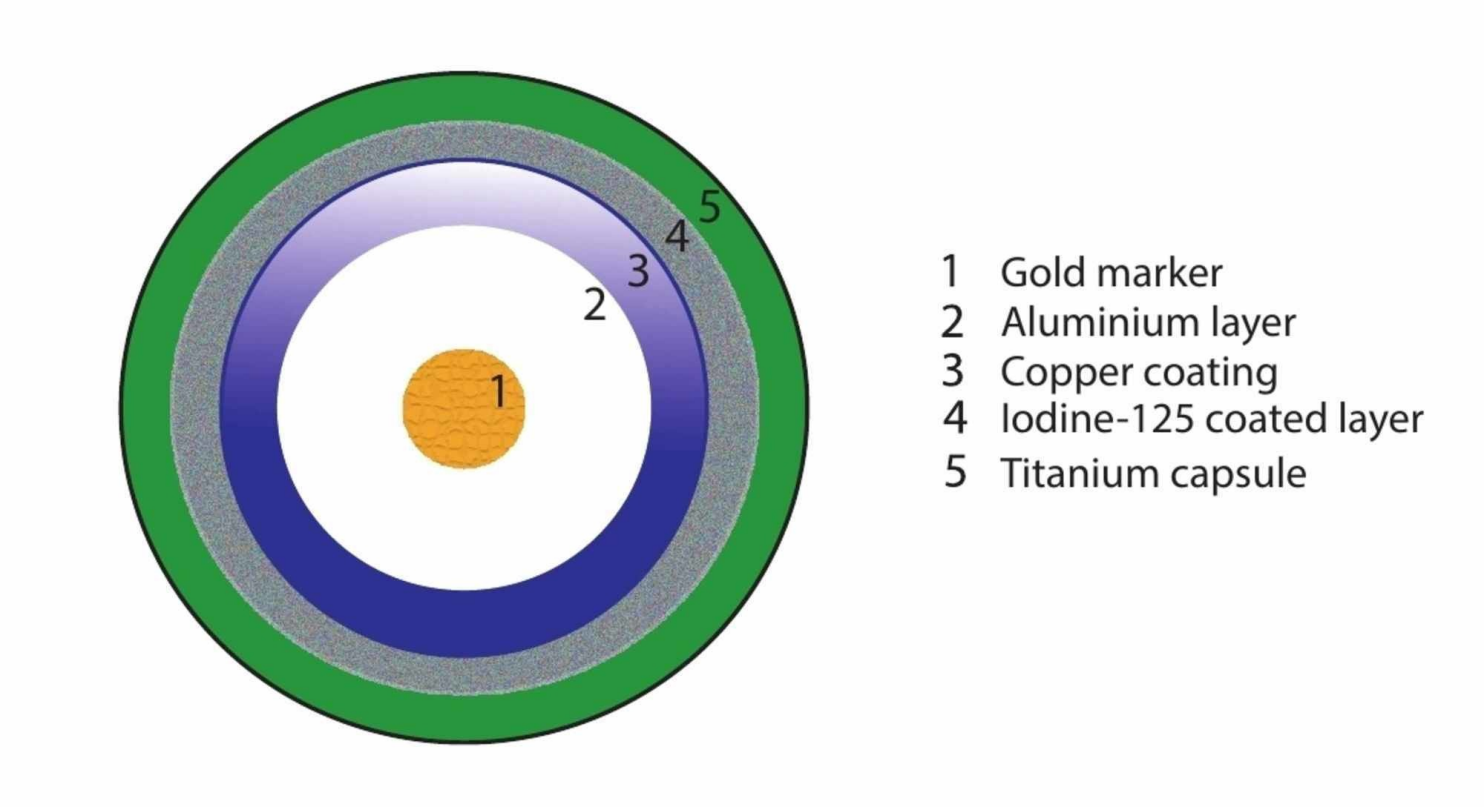
Main components of a radioactive iodine-125 seed. Adapted from Straver et al. [6]

A 63-year-old patient was referred to our team for the surgical management of a squamous cell carcinoma of the skin with axillary metastases. Apart from obesity (i.e., BMI=34) and a history of lower back skin burns, the patient did not have any previous medical history. Prior to referral, the primary lesion had been resected with negative margins. The carcinoma was a poorly differentiated 5 mm thick ulcerated lesion, measuring 6 cm in diameter, located in the patient’s right dorsolumbar region. The tumor did not extend beyond the subcutaneous fat. Two months after the resection of the primary tumor, plastic surgery performed a thoracodorsal perforator flap and two metastatic axillary lymphatic nodes were found on pathological analysis. A postoperative PET/CT revealed the presence of a hypermetabolic axillary lymph node on the right side (Figure 1A). A biopsy of the axillary node confirmed the diagnosis of carcinoma and a metallic marker was inserted. Due to increased risks of side effects, related to the patient history of burns, radiation oncology decided against adjuvant radiotherapy. After presentation of the case at the multidisciplinary cancer conference, we proceeded with a bilateral axillary lymph node dissection. Surprisingly, neither metastatic node nor marker was retrieved in the right axilla on pathological analysis, and two nodes out of 10 were positive in the left axilla. We concluded that the previously proven metastatic node in the right axilla had been missed during the dissection. A postoperative ultrasound confirmed the presence of a 1.1 cm lymph node, containing a metallic marker, localized posteriorly, near the latissimus dorsi. Therefore, an I125 seed (Figure 2) was inserted under ultrasound guidance. The metastatic node was resected using radioactive seed localization. A small incision in the muscle fibers of the latissimus dorsi was needed to access the node. No subsequent recurrence was diagnosed at 21 months of follow-up.

Case 2

An 82-year-old patient was referred by his general practitioner for the surgical management of a recently diagnosed malignant melanoma on his left shoulder. The tumor was an ulcerated mass with a Breslow thickness of 5.9 mm and 16 mitoses per mm 2. A preoperative PET/CT demonstrated the presence of an ipsilateral hypermetabolic retropectoral lymph node measuring 2.9 cm (Figure 1B). A radioactive seed was inserted in the radiology department into this nonpalpable node. We performed local excision with 2 cm margins, together with the targeted removal of the seed node and a sentinel lymph node biopsy. Both Technetium-99 and methylene blue were used during the procedure. In addition to the seed node, five distinct sentinel nodes were resected. On the final pathology report, a 3 cm metastatic invasion was found in the seed node. The patient was followed by medical oncology in another center.

Case 3

**Figure 3:**
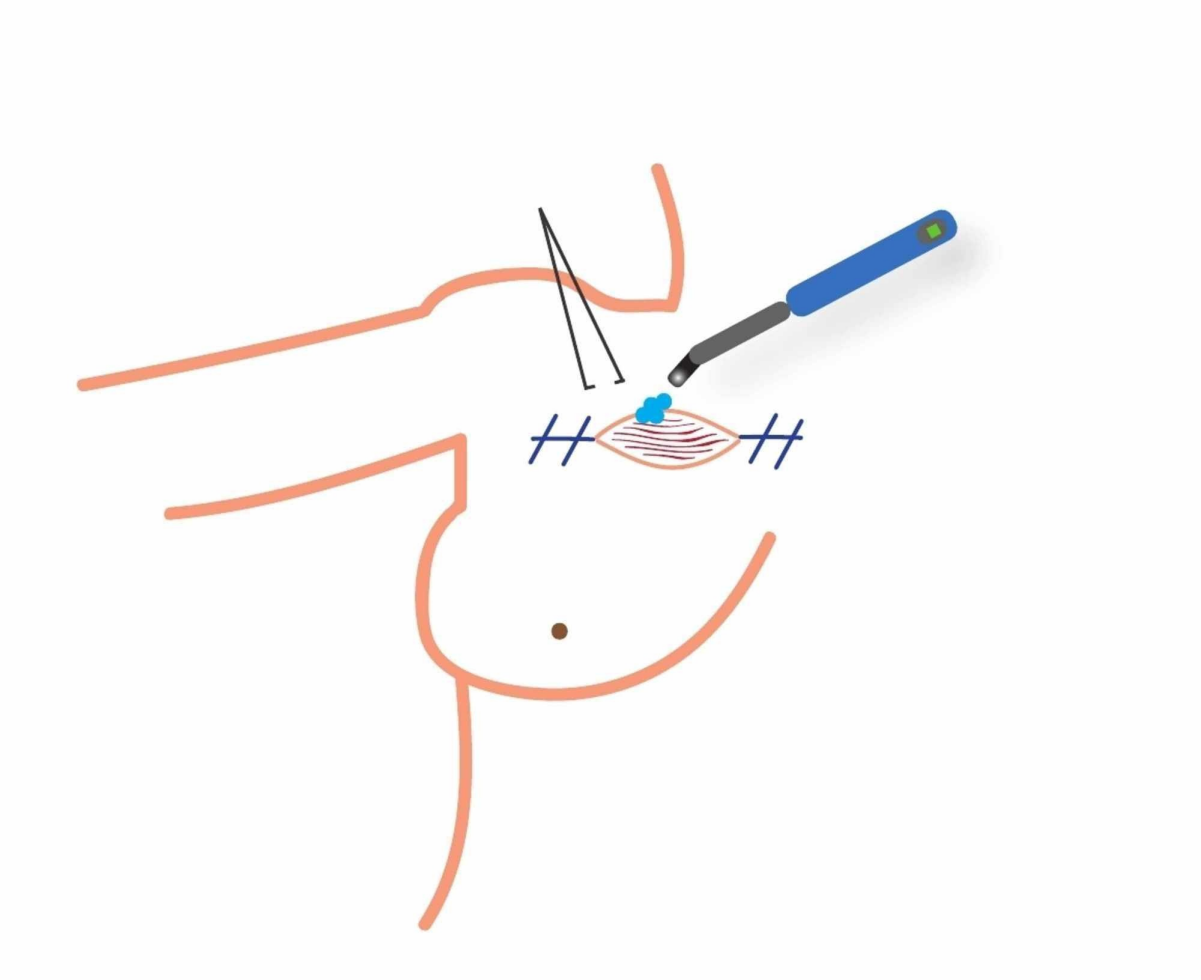
Radioactive seed localization using a transpectoral approach.

A 41-year-old patient was seen in consultation for a second opinion concerning a positive retropectoral node. The patient had been previously operated for a triple-positive ipsilateral invasive ductal carcinoma (IDC) following neoadjuvant chemotherapy. The tumor was T3N3b and an abnormal internal mammary node was detected on initial imaging. An axillary lymph node dissection was performed, and metastases were detected in three out of nine harvested nodes. Sixty-two Gy of radiotherapy were administered in the adjuvant setting on the left anterior thorax, the left axilla, and the supraclavicular area. A bilateral prophylactic salpingo-oophorectomy was realized during the following year and the patient received endocrine therapy. Eighteen months later, a suspicious 1 cm retropectoral lymph node was detected on diagnostic chest CT performed to rule out pulmonary embolism in the context of acute dyspnea. A biopsy of the lymph node revealed the presence of breast carcinoma. There was no evidence of local recurrence or other distant metastatic sites on PET/CT (Figure 1C). The patient and the oncology team opted for a level III lymphadenectomy with the targeted resection of the metastatic node. The node was accessible for I125 seed insertion under ultrasound guidance. During surgery, a frozen section and radiography of the specimen both confirmed the successful removal of the positive seed node. No other nodes were found in the specimen on pathological analysis. The patient received adjuvant endocrine therapy and no recurrence was noted at 25 months of follow-up. Figure 3 illustrates a transpectoral approach.

Case 4

A 66-year-old patient was referred to our academic hospital center for breast cancer recurrence in level I, II, and III left axillary lymph nodes. The patient’s past medical record included disseminated lupus erythematosus. Three years prior, the patient was diagnosed with a T2N1a invasive ductal carcinoma in the left breast. The tumor was estrogen receptor (ER)-positive and human epidermal growth factor receptor 2 (HER2)-negative. Due to lupus skin involvement precluding radiotherapy, a modified radical mastectomy was performed followed by adjuvant chemotherapy and endocrine therapy. Two years later, several enlarged ipsilateral axillary lymph nodes were found on a thoraco-abdominal CT, prescribed to investigate increased carcinoembryonic antigen (CEA) levels. A PET/CT and a biopsy confirmed a recurrence (Figure 1D). An axillary MRI demonstrated multiple abnormal nodes in level I, one 3.2 cm abnormal node in level II and one 1.2 cm node in level III. Before referral to our institution, the patient had a partial radiological response to systemic treatment including an aromatase inhibitor and a CDK4/6 inhibitor.

On a second PET/CT, obtained nine months later, a decrease in size of the axillary lymph node was noted with no distant metastases. An axillary lymph node dissection (ALND) with radioactive seed localization was performed. An I125 seed and a clip were implanted in the level III lymphadenopathy, located posterior to the pectoralis major. Due to fibrosis, the axillary lymph node dissection was arduous. After opening the pectoralis major muscle, the seed node, as well as three distinct enlarged nodes were removed. Five of 13 lymphatic nodes were metastatic on pathological analysis, including the targeted node. The patient continued receiving endocrine therapy and did not develop any recurrence at 23 months of follow-up.

Case 5

A 53-year-old patient was diagnosed with invasive ductal carcinoma and a biopsy proven nonpalpable metastatic axillary lymph node. Partial mastectomy and sentinel lymph node biopsy were performed. However, the metallic clip implanted before surgery to mark the metastatic node was not detected on the intraoperative radiography of the specimen. Pathological analysis revealed two micrometastases in one of the three resected nodes. A second axillary ultrasound was undertaken postoperatively. The metastatic node and the metallic clip were both identified. A radioactive iodine-125 seed was inserted in the lymph node to help perform a subsequent targeted lymphadenectomy. The surgery was successful. The patient received adjuvant chemotherapy (i.e., doxorubicin, cyclophosphamide, and paclitaxel) followed by locoregional radiotherapy and endocrine therapy. The patient remained free of recurrence at 43 months of follow-up.

## Discussion

This article illustrates how radioactive seed localization of occult axillary lesions can help overcome important clinical challenges (Table 1). In this case series, we used radioactive seeds to retrieve occult axillary lesions, including one case where a positive node would have been missed post ALND. Despite the fact that there is no evidence of improved breast cancer prognosis with the removal of regional metastases, the decision to proceed with this technique can be the best alternative for patients in which systemic chemotherapy or hormonal therapy is not an option.

**Table 1:** Cases summary. I^125 ,^ iodine-125; ALND, axillary lymph node dissection.

Case ID	Primary tumor	Reason for I^125^ seed use	Complications
Case 1	Squamous cell carcinoma	Failure to remove a level I metastatic node during ALND	Absence
Case 2	Malignant melanoma	Targeted removal of a retropectoral hypermetabolic node	Absence
Case 3	Invasive ductal carcinoma	Isolated proven positive retropectoral lymph node	Absence
Case 4	Invasive ductal carcinoma	Level III axillary recurrence	Absence
Case 5	Invasive ductal carcinoma	Level III and supraclavicular recurrence	Absence

Although axillary radioactive seeds have been used for diagnostic purposes in malignant melanoma [10], to our knowledge, our group is the first to report radioactive seed localization to achieve a complete axillary dissection in malignant melanoma. In a previously published prospective study, radioactive seeds were placed in suspicious axillary, cervical, and supraclavicular nodes in patients with occult primary tumors. One of these patients was subsequently diagnosed with malignant melanoma [10].

Other groups described the use of radioactive seeds to resect metastatic lesions from malignant melanoma [11-12]. Fleming et al. reported a case in whom a radioactive seed was used to successfully resect subcutaneous nonpalpable in-transit lesions [11]. The lesions which were in the patient’s right lower extremity, had been detected on PET/CT and further characterized on a subsequent MRI before seed insertion. Dissanayake et al. reported using an iodine-125 seed to remove a nonpalpable 4 mm posterior chest wall melanoma metastasis [12]. Furthermore, human serum albumin labeled with Technetium-99 was used in another series to perform axillary and inguinal dissection in 12 patients diagnosed with melanoma [13]. In all cases, the purpose of the procedure was to remove nonpalpable suspicious nodes. However, the technique requires scintigraphy to assess the accuracy of the injection and the successful migration of the radiotracer is not guaranteed. Radioactive seed localization seems to be a more reliable technique to successfully identify and excise the targeted node.

Even though radioactive seeds have been inserted for brachytherapy purposes in oral squamous cell carcinoma [14], this technique has not been described in squamous cell carcinoma of the skin.

Various approaches have been published in breast cancer, including wire-guided lymphadenectomy, intraoperative ultrasound [5, 15], and radioactive seed localization [7, 9, 16-17]. Nevertheless, most of the published methods aim to identify a previously proven metastatic node after neoadjuvant chemotherapy in patients with complete axillary clinical response. The main advantage is to spare patients the morbidity associated with ALND. We aimed to report on the usefulness of this technique in unconventional situations.

Compared to wire-guided localization surgery, the localization of axillary nodes using radioactive seeds has several technical advantages and increases the likeliness to retrieve the suspicious node compared to sentinel lymph node biopsy alone. During this procedure, the surgeon receives continuous feedback resulting in a reduction of the extent of the dissection, with minimal morbidity. Radioactive seed localization also helps the surgeon when scar tissue is present with increased risk of neurovascular trauma.

Furthermore, radioactive seeds are associated with less discomfort and displacement than guide wires [2-3]. Guide wires are known for specific complications, such as transection and kinking, and are associated with organizational constraints, as patients are required to have surgery on the day the wire is inserted in radiology.

Another option is the positioning of a metallic clip at the same time as the node biopsy, followed by the ultrasound guided removal of the clipped node [18]. However, the operating room needs to be fully equipped in order to perform this procedure and the surgeon needs to have significant experience with intraoperative ultrasonography.

## Conclusions

With remarkable advances in medical imaging, the frequency of the detection of occult lymph node metastases has increased over the past years. This has revolutionized the practice of surgical oncology and positions the surgeon at the center of clinical enigmas regarding the therapeutic management of these patients. Flexibility is required in order to make the most appropriate decisions and to act in accordance with patients’ wishes. This case series shows that radioactive iodine-125 seeds can become key tools in the surgical management of complex oncological cases, particularly in breast cancer, malignant melanoma, and squamous cell carcinoma of the skin.
